# Purely one-dimensional bands with a giant spin-orbit splitting: Pb nanoribbons on Si(553) surface

**DOI:** 10.1038/srep46215

**Published:** 2017-04-06

**Authors:** Marek Kopciuszyński, Mariusz Krawiec, Ryszard Zdyb, Mieczysław Jałochowski

**Affiliations:** 1 Institute of Physics, Maria Curie-Sklodowska University, Lublin, 20-031, Poland

## Abstract

We report on a giant Rashba type splitting of metallic bands observed in one-dimensional structures prepared on a vicinal silicon substrate. A single layer of Pb on Si(553) orders this vicinal surface making perfectly regular distribution of monatomic steps. Although there is only one layer of Pb, the system reveals very strong metallic and purely one-dimensional character, which manifests itself in multiple surface state bands crossing the Fermi level in the direction parallel to the step edges and a small band gap in the perpendicular direction. As shown by spin-polarized photoemission and density functional theory calculations these surface state bands are spin-polarized and completely decoupled from the rest of the system. The experimentally observed spin splitting of 0.6 eV at room temperature is the largest found to now in the silicon-based metallic nanostructures, which makes the considered system a promising candidate for application in spintronic devices.

A search for new materials which could speed up the development of electronics and computing capabilities becomes an important area of research in physics, chemistry and materials science. Among various solutions spintronics is the one which offers such ability. Beside the charge of an electron it makes use of its spin giving an additional degree of freedom for carrying bits of information^1^. For this purpose spin-split electronic bands at the Fermi level (*E*_*F*_) are necessary with the splitting at room temperature large enough to prevent mixing of electrons with the opposite spins in transport processes.

Usually the spin degeneracy of electron bands can be lifted either by the magnetic field (external or internal) or by strong spin-orbit interaction. The latter effect can be observed in a bulk crystal (Dresselhaus effect) and/or at its surface or interface (Rashba-Bychkov (RB) effect). While the Dresselhaus effect is entirely determined by intrinsic properties of a crystal the RB effect can be easily modified by various means as recently shown in a number of reports^2^,^3^,^4^,^5^,^6^,^7^,^8^,^9^,^10^,^11^,^12^,^13^. Unfortunately, in most cases the spin splitting is to small to be utilized in spintronic devices. If it is large, then the spin-split bands usualy are fully occupied/unoccupied^2^,^5^,^6^,^10^,^11^,^14^,^15^,^16^ or the substrate is metallic appending huge unpolarized currents during spin transport^2^,^3^,^4^. Thus, beside large spin splitting of metallic states at the Fermi level a desired system requires a semiconducting substrate, with silicon being the best choice as widely used in technology.

Among various systems studied up to now and fulfilling some of the above criteria are semiconductors with their surface states Bi_2_Se_3_^17^, BiTeI^9^, metal overlayers on semiconducting substrates as Pb/Ge^7^ or Si(557)-Pb^18^, reconstructions of Si(111) induced by different elements^12^,^19^ and vicinal silicon surfaces ordered by the presence of Au atomic chains^8^,^20^,^21^,^22^,^23^. Nanostructures of the latter group have one-dimensional (1D) character and as such they can reveal an extra advantage – a strong suppression of spin-orbit scattering^24^,^25^. However, in addition to quite low spin splitting, in most cases of Si reconstructions a strong interaction between adatoms and the surrounding atoms of the silicon matrix occurs which is an undesirable effect (mixing of spin-polarized and unpolarized electrons).

In this Letter we report on novel electronic and spin structures of Pb nanoribbons on a Si(553) surface, where the drawbacks mentioned above are overcome. The Pb nanoribbons have one-dimensional character with metallic surface states perfectly decoupled form the electronic structure of silicon substrate. These states reveal giant Rashba spin-orbit splitting at *E*_*F*_ of more than 0.6 eV with Δ*k*_*F*_ = 0.2 Å^−1^, as revealed by the spin- and angle-resolved photoemission spectroscopy [(S)ARPES] measurements, and is the largest one observed up to now for metallic bands in the silicon-based systems at room temperature. According to density functional theory (DFT) calculations such giant spin splitting is caused by the unique arrangement of Pb atoms on each terrace of the surface, in which Pb atoms form a strained, strongly distorted hexagonal lattice. This results in the appearance of a strong anisotropy in the charge distribution across the steps, and a large spin splitting of the surface bands, including the appearance of an out-of-plane component of the spin polarization.

## Results and Discussion

The deposition of 1.3 ML Pb on Si(553) surface results in a Pb wetting layer which upon annealing makes the underneath Si surface ordered with perfectly regular distribution of monatomic steps. The crystallographic structure and morphology of the Si(553)-Pb surface determined by RHEED and STM experiments and DFT calculations have been reported in ref. 26. The Pb atoms form nanoribbons on each Si(111) terrace and each nanoribbon consists of five Pb atomic chains. A model of the surface predicts no intermixing between Pb and Si atoms compared to Si(553)-Au^26^,^27^. For more details about experiment and model calculations see Supplementary Information.

**Figure 1:**
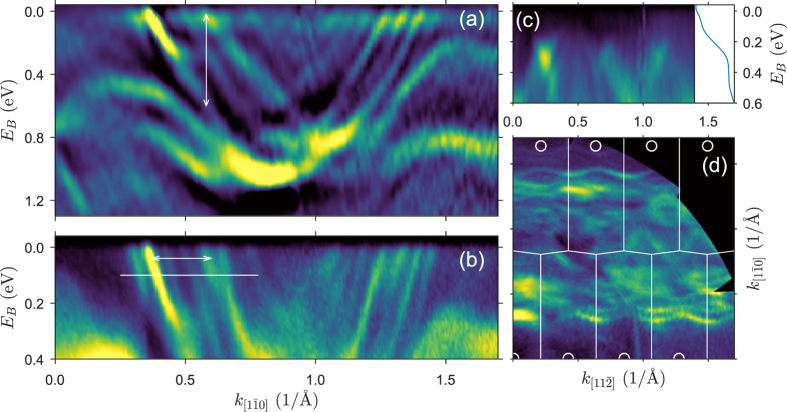
Electronic structure of Si(553)-Pb. Photoemission intensity (2nd derivative) maps of the binding energy vs wave vector parallel (**a**), (**b**) and perpendicular (**c**) to the step edges. The double arrows in (**a**) and (**b**) indicate oppositely polarized bands and the white line in (**b**) - energy range at which spin-resolved measurements have been done. The inset in (**c**) shows energy distribution curve obtained from the photoemission intensity map across the steps. (**d**) The Fermi surface of Si(553)-Pb with the edges and centers of the surface Brillouin zones shown as thin, white lines and circles, respectively. Zero of the binding energy denotes the Fermi level.

The electronic structure of the Si(553)-Pb surface in the form of photoemission intensity (second derivative) maps recorded in the 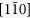 direction, i.e. parallel to the step edges are shown in Fig. 1(a,b). The band structure shows several 1D parabolic-like bands with a highly intense minimum around 1 eV below *E*_*F*_ located around the 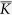 point of the surface Brillouin zone, Fig. 1(a). A similar but single band has been observed in the electronic structure of Pb chains grown on the Si(553)-Au^28^ and Si(335)-Au^29^,^30^ surfaces and has been reported to be the 6*p* band of lead. These bands are shown in the high resolution photoemission map close to the Fermi level, Fig. 1(b).

Although the Pb coverage exceeds 1 ML, the surface reveals a band gap in the direction perpendicular to the steps, Fig. 1(c), which indicates the 1D character of the bands. The inset of Fig. 1(c) presents energy distribution curve obtained after integration of the photoemission intensity over the entire Brillouin zone across the steps. It shows slowly increasing background of the secondary electrons below *E*_*F*_ and significant intensity drop at about 110 ± 10 meV. The 1D character of the surface is also visible in the Fermi surface, Fig. 1(d), where the bands are elongated in the 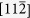 direction. Their wavy shape, however, indicates the weak interaction between Pb chains.

**Figure 2:**
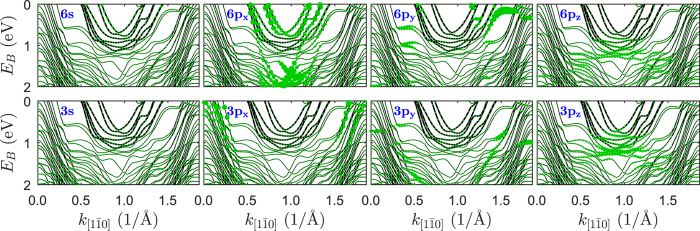
Calculated electronic structure of Si(553)-Pb. Contribution of different orbitals, denoted by a diameter of circles, to the band structure of Si(553)-Pb. Top row - Pb, bottom row - Si.

According to the DFT calculations the band structure along the nanoribbons reveals six parabolic-like bands crossing the Fermi energy around the 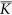 point of the surface Brillouin zone similarly as in the experiment, Fig. 2, (the corresponding bands are denoted as the thicker lines). It is important to note that the DFT calculations of the electronic structure have been performed within reduced unit cell geometry model, Fig. S1 of Supplementary Information and Methods section. Within that model the Pb atoms are uniformly distributed on the terrace and form an almost ideal hexagonal lattice with the unit cell of 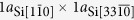. In fact, the hexagonal lattice is strongly distorted and the distances between Pb atoms in the 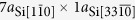 unit cell are not uniform and vary between 3.04 and 3.60 Å, Fig. S1 of Supplementary Information. Applying the full unit cell geometry model in the DFT calculations, which includes more than 300 atoms, gives a huge number of folded bands and makes obtained results useless. Thus, in the following, the calculated electronic structure is based on the reduced unit cell geometry model. Therefore, we do not expect a perfect agreement between the band structures obtained with DFT and ARPES. Nevertheless, such calculations should give some clues regarding the nature and origin of the measured electronic structure.

The results presented in Fig. 2 have been obtained for the Pb lattice constant of 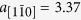 Å, which is the average Pb-Pb distance in this system found previously^26^. To shed additional light on the electronic structure of the surface we have also made calculations for other lattice constants, Fig. S2 of Supplementary Information. In each case there are three pairs of parabolic-like bands crossing the Fermi level around the 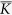 point of the surface Brillouin zone and the bands are introduced by Pb nanoribbons. Positions and shapes of the bands strongly depend on the lattice constant along 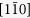, as Fig. S2 illustrates. It turns out that the experimental data are reproduced best in calculations with 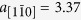 Å.

Interestingly, the considered bands are derived almost entirely from the 6*p*_*x*_ orbitals of lead as indicated by the diameter of circles in Fig. 2. The contribution of other orbitals is negligible, what suggests that electrons in these bands are completely decoupled from the rest of the system. The most striking feature is the lack of significant contribution of 6*p*_*y*_ orbitals (y direction is perpendicular to the nanoribbons) to the considered bands. It is apparently responsible for the existence of the energy gap across the steps. The very weak interaction between Pb and the substrate is owing to the hybridization of corresponding *p*_*z*_ orbitals, as Fig. 2 illustrates. Thus we can regard Pb nanoribbons as one-dimensional objects which are very well decoupled from the substrate.

**Figure 3:**
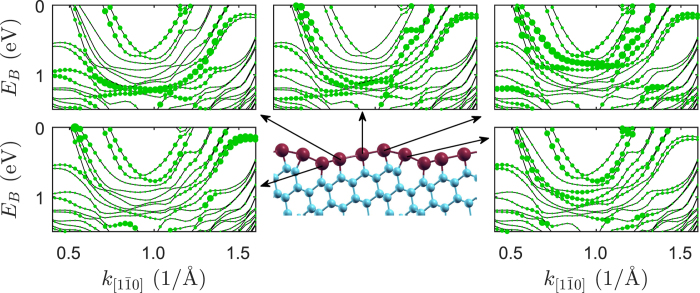
Calculated electronic structure of Si(553)-Pb. Band structure of Si(553)-Pb projected on different rows of Pb atoms.

The weak contribution of the 6*p*_*y*_ orbitals can also be observed when the band structure is projected onto individual Pb chains, Fig. 3. Namely, each row of Pb atoms contributes to the band structure located in a different part of the Brillouin zone. The hybridization of these states between neighboring Pb rows is weak, thus one can think of this system as of the set of independent Pb chains rather than Pb nanoribbons. However, some of the bands spread over the whole nanoribbon with varying contribution from different chains. Bands V and VI show such behavior, which indicates that Pb chains are not completely independent. This also is supported by ARPES data, where the Fermi surface shows wavy shapes. On the other hand, a pair of bands I-II and III-IV are localized on particular chains. Thus we deal with two different sets of bands: localized on atomic chains and delocalized. Similar conclusions can be made looking at the charge distribution along the Pb chains for each considered band, Fig. S3.

The DFT calculation of the band structure also corroborate the presence of the energy gap across the steps. The results are shown as thin lines superimposed on the photoemission map, Fig. S4(a). In addition to the lines the circles represent contribution of lead atoms to the bands. The clear periodicity in the band structure along this direction stems from the regular step distribution on the surface. Beside Pb-induced bands, which are located about 110 meV below the Fermi level, the calculations give also several non-dispersive bands coming from Si. One of them is located about 10 meV below *E*_*F*_, Fig. S4(a). Its absence in the experimental data can be due to a small spectral density of this surface state superimposed on the observed relatively high background of secondary electrons. It also must be kept in mind that the reduced unit cell geometry model used in the band structure calculations does not describe properly real surface thus the perfect agreement between the results of calculations and experiment is not expected.

**Figure 4:**
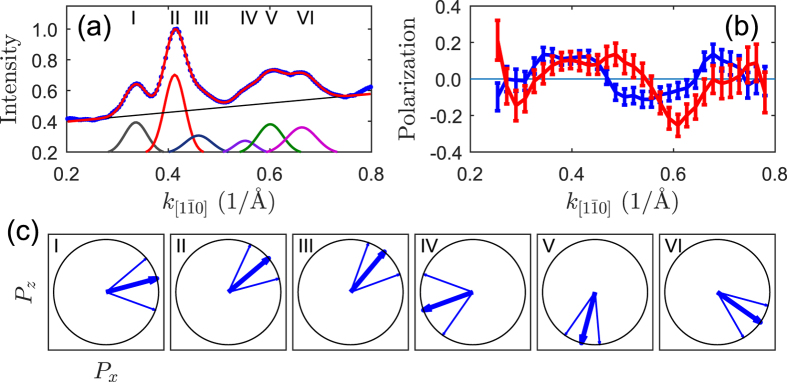
Results of the SARPES experiments. (**a**) Spin-averaged photoemission intensity (blue dots) of the bands indicated in Fig. 1(b) together with a fit (red line). The fitted peaks and background are shown as separate curves below experimental data. (**b**) In-plane (blue) and out-of-plane (red) components of the polarization vector. (**c**) Direction of the polarization vector of the considered bands. P_z_ and P_x_ are parallel to the 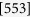 and 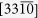 directions, respectively. The number in each diagram denotes corresponding intensity peak (band) shown in (**a**).

This unusual 1D strongly metallic multi-band structure makes this surface a very unique system. This becomes even more exciting when noticing that the close lying bands crossing the Fermi level resemble the spin-split bands observed in the case of Si(557)-Au and Si(553)-Au surfaces^8^,^22^,^23^. The spin-split bands hypothesis becomes more plausible bearing in mind that Pb atoms feature strong spin-orbit interaction (*Z* = 82) and the low dimensionality of a system can increase this interaction^31^. In addition, the Rashba-split bands have been observed in a number of Pb structures on different substrates^4^,^13^,^18^,^32^,^33^,^34^. Indeed, the spin-resolved ARPES measurements reveal a clear asymmetry between photemission intensity recorded with two channels of the Mott detector. The measurements have been done for the bands indicated by the white line in Fig. 1(b). The spin-averaged photemission intensity distribution of that region is shown in Fig. 4(a) (blue dots) together with the fit (red line) and six peaks (corresponding to six bands), and the background (straight line) used to fit to the experimental data.

The asymmetry in the photoemission intensity of each band measured with two channels of the Mott detector results in the polarization of the bands as shown in Fig. 4(b). Apart from the in-plane polarization component (blue) which is perpendicular to Pb nanoribbons a comparable in magnitude out-of-plane component (red) also appears. The spin-resolved photoemission intensity curves of both components are shown in Fig. S5. Further analysis of the spin-resolved data has been performed according to the recipe given in ref. 33. The obtained directions of the polarization vector of the bands are presented in Fig. 4(c). Clearly, the polarization vector of each band has a significant out-of-plane component. Within the limit of error bars (denoted as the neighboring thin blue lines) there is a clear correlation between the direction of polarization of the bands. The polarization vectors of the first three bands are almost parallel to each other. Simultaneously, they are roughly antiparallel to the polarization of the forth and fifth bands. Apparently, the polarization of the sixth band does not follow this rule. In addition, it seems to be a counterclockwise rotation of the polarization vector and increasing out-of-plane component going from the first to the last band.

Because of the rotation of the polarization vector and large error bar it is not possible to unambiguously assign oppositely polarized bands and determine a value of spin splitting. However, thinking of the Rashba spin-split bands, it means the bands with opposite spin polarization, several combination of the bands can be considered e.g. (I and IV), (I and V), (I and VI) and analogous combinations for the bands II and III. Considering the following pairs (I and IV), (II and V) and (III and VI) as the Rashba spin-split bands the values of Δ*k*_*F*_ = 0.2 Å^−1^ and more than 0.6 eV at the Fermi level have been obtained. All other combinations of the oppositely polarized bands, like e.g. (I and V) or (I and VI), would give much larger values of the spin splitting parameters for at least one pair of the bands. Thus the presented above choice of the bands gives the smallest set of values from possible combinations.

**Figure 5:**
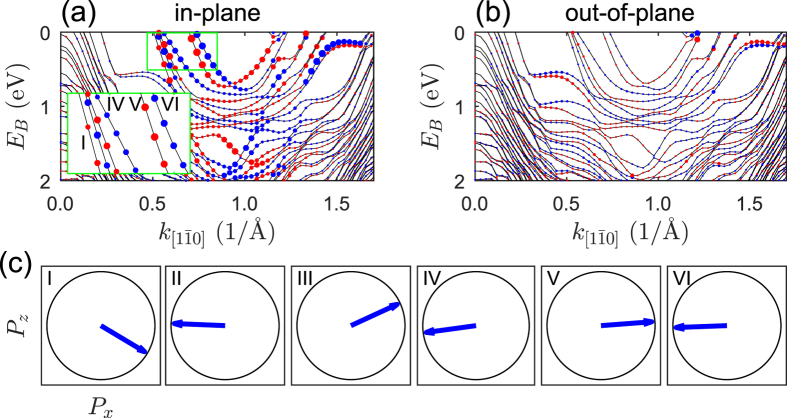
Calculated electronic structure of Si(553)-Pb. Band structure of Si(553)-Pb calculated with 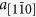 = 3.37 Å (**a**,**b**). The oppositely polarized bands are indicated by the red and blue circles. A diameter of the circles determines a value of the corresponding component of the polarization vector. (**c**) Direction of the polarization vector of three pairs of bands determined at *E*_*B*_ = 0.09 eV. The inset in (**a**) presents a part of 1D bands which are of interest for the determination of the polarization vector direction shown in (**c**). The number in each diagram denotes corresponding band indicated in the inset of (**a**).

According to the DFT calculations the surface Pb bands are split, and the splitting originates from the spin-orbit interaction. Interestingly, the polarization vectors have both in-plane, Fig. 5(a), and out-of-plane, Fig. 5(b), components, similar as in the experiment (see Fig. 4). A potential gradient along the direction perpendicular to the surface is responsible for the appearance of the in-plane component of the polarization vector. On the other hand, the Pb-row-projected electron states located in different parts of Brillouin zone are responsible for the asymmetry in partial charge distribution within the surface, thus for the out-of-plane spin polarization component.

**Figure 6:**
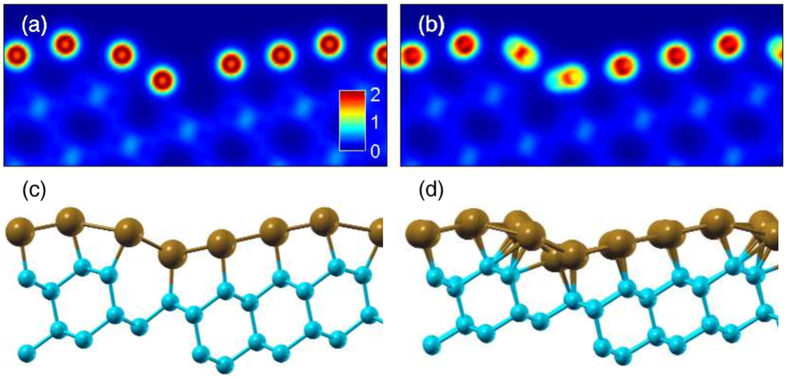
Calculated charge distribution around Pb atoms. Charge distribution (**a**) in the reduced unit cell geometry (with 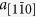 = 3.37 Å) and (**b**) in the full unit cell geometry models of the Si(553)-Pb surface. A color code denotes charge density in electrons/bohr^3^ units. A side view (along 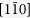) of the reduced (**c**) and full (**d**) unit cell geometry models. The charge density is averaged over the whole unit cell along 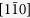. The brown (light blue) circles denote Pb (Si) atoms.

The existence of the component of the polarization vector perpendicular to the surface can also be explained by an asymmetric charge distribution around Pb atoms, Fig. 6. In the case of the reduced unit cell geometry model the asymmetry in the charge distribution is very small, Fig. 6(a), thus the obtained out-of-plane component is small too. However, the corresponding calculations in the full unit cell geometry model of Si(553)-Pb show significant charge asymmetry in the plane of the surface, Fig. 6(b). This is the result of the nonuniform distribution of Pb atoms, and should lead to the appearance of a strong perpendicular component of the polarization as observed in other systems^23^. Indeed, the experiment also gives the out-of-plane polarization component (Fig. 4) much larger than the one predicted by the reduced unit cell geometry model, Fig. 5(b,c). Similar calculations performed along the steps show quite significant asymmetry, too, Fig. S6. However, as the asymmetry in the charge distribution and the resulting electric field is parallel to the 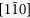 direction it could contribute to the out-of-plane component of the polarization vector only if the electron have moved in the perpendicular direction, i.e. 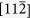.

However, there is a significant difference between experiment and theory in the polarization vector direction of each band. While the experimental data show first three bands with almost parallel polarization, Fig. 4(c), the reduced unit cell geometry model usually gives an alternate arrangement of the direction of the polarization vector of the considered bands, Fig. 5(c). The situation is similar for larger lattice constants (Fig. S2(b–d)). However, the directions of polarization vary substantially with Pb-Pb distance. Interestingly, for the shortest lattice constant considered here the spin polarization starts to deviate from this picture, and the polarization vectors are no longer antiparallel in the pair of bands. Thus the observed difference between experiment and theory may be attributed to the unusual arrangement of Pb atoms with varying inter-atomic distance in a nanoribbon. Unfortunately, without DFT calculations performed within the full unit cell geometry model it is not possible to make direct comparison of the calculated and experimental electronic structures and receive more conclusions regarding the Rashba spin-split states.

Apart from the appearance of the out-of-plane component of the polarization vector, the in-plane asymmetric charge distribution around nuclei of heavy atoms is also known to strongly enhance the spin splitting of the surface bands^35^. Such asymmetry of the charge distribution is already predicted for the full unit cell geometry model shown in Fig. 6(b) and support the giant spin splitting observed in the experiment.

The rotation of the polarization vector from in-plane to out-of-plane direction with increasing wave vector value might be related to changes in orbital character of the bands. Although we cannot directly check this hypothesis by the present DFT calculations in the full unit cell geometry model due to a huge number of folded bands, similar argumentation has explained the rotation of the polarization vector observed in the Tl/Si(111) system^16^.

A giant splitting of electron bands has been observed in other systems too, however, either for fully occupied/unoccupied bands e.g. Pt/Si(110)^11^, Bi/Si(111)^5^,^14^, Tl/Si(111)^16^ or for surfaces of metals^2^. Systems which combine a semiconducting substrate and surface bands crossing the Fermi level are much less common. These include Pb/Ge(111)^7^, different reconstructions of Si^12^,^19^, vicinal silicon surfaces with Au-induced ordering^8^,^22^,^23^ and Si(557)-Pb^18^. The latter system is similar to that reported here, however, Δ*k*_*F*_ = 0.2 Å^−1^ is observed only at temperatures below 78 K where Fermi nesting occurs^36^. At this temperature Pb-induced refacetting from (557) (above 78 K) to (223)-oriented facets (below 78 K) occurs. It means that below 78 K the system is not homogenous - there are (223) facets which reveal 1D character and wide (111) terraces with 2D character. Thus, the Si(553)-Pb system reveals the largest observed band splitting in both wave vector and energy scale at room temperature. In addition, its crystallographic structure is stable up to around 550 K and the exact Pb coverage is not so crucial as in the case of Si(557)-Pb^18^. The other advantage is the well-defined 1D character of the system even at room temperature, thus presumably strongly reduced spin-orbit scattering. However, this is an open issue and requires further investigations.

From the point of view of spin-dependent transport phenomena and potential applications the most important quantities are those determined at the Fermi level. The large spin splitting of the bands indicates strong spin-orbit coupling which in turn suggests possible applications. Taking the present value of Δ*k*_*F*_ = 0.219 Å^−1^ one can calculate a dephasing length which defines the distance after which spins precess by 180 degrees and can be considered as a gate length in a spin field effect transistor^37^. In the case considered the length is 1.4 nm which is among the shortest ones reported to now for spintronic devices. Together with the energy separation of the spin-split bands at the Fermi level (0.6 eV) this makes this system a promising route for developing spintronic devices operating at room temperature.

In summary, we have studied electronic and spin structures of the vicinal Si(553) surface with a regular distribution of steps over the macroscopic size area stabilized by the presence of Pb overlayer. The system exhibits a strongly metallic and one-dimensional character of electron bands even at room temperature. The Pb-induced surface bands are spin-polarized due to the spin-orbit interaction. The observed giant spin splitting of the bands is caused by the unusual strained arrangement of the Pb atoms on the terraces and resulted anisotropic electron charge distribution around Pb nuclei. Such giant spin splitting leads to very short dephasing length which can be of importance in spintronic technology. The present system fulfills the most important criteria to be utilized in spintronic devices and can safely work at room temperature still having giant spin-split bands at the Fermi level. The spin-split surface bands are perfectly decoupled from the substrate assuring pure spin currents preventing their mixing with unpolarized substrate currents.

## Methods

The Pb nanoribbons on Si(553) have been prepared by deposition of 1.3 ML (monolayers) of Pb on freshly flashed vicinal Si. 1 ML is defined as density of atoms in a half of the bulk terminated Si(111) bilayer. After deposition the Pb layer was annealed at 250 °C. Details can be found in refs. 26,38. The SARPES measurements have been performed with a high brightness helium lamp (unpolarized He I line) and a high resolution hemispherical analyzer (Phoibos 150) with a two-dimensional MCP electron detector and 12 bit CCD camera, Fig. S7. For the spin-resolved detection a 25 keV Mott detector was used. The energy and angular resolutions were set to 30 meV and 0.2° for the angle-resolved and 100 meV and 0.5° for the spin-resolved measurements, respectively.

The calculations were performed within Perdew-Burke-Ernzerhof (PBE)^39^ generalized gradient approximation (GGA) to density functional theory using projector-augmented-wave potentials, as implemented in VASP (Vienna ab-initio simulation package)^40^,^41^. A plane wave basis set with energy cutoff was set to 340 eV. We have considered two unit cells: the full unit cell geometry 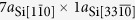 (in short 7 × 1, discussed in ref. 26) and the reduced unit cell geometry 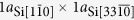 (1 × 1). Both structures are shown in Fig. S1.

The corresponding Brillouin zones were sampled by a 2 × 2 × 1 and 7 × 2 × 1 Monkhorst-Pack k-points grids^42^, respectively. In structural relaxation all the atomic positions, except the bottom layer, were fully relaxed until the maximum force in any direction was less than 0.01 eV/Å. The Si atoms in the bottom layer were fixed at their bulk ideal positions and saturated with hydrogen atoms. The band structure calculations were performed in the reduced unit cell geometry taking into account spin-orbit interaction. The charge density distributions were calculated using the SIESTA code^43^,^44^,^45^,^46^ with a similar set of parameters.

## Additional Information

**How to cite this article**: Kopciuszyński, M. *et al*. Purely one-dimensional bands with a giant spin-orbit splitting: Pb nanoribbons on Si(553) surface. *Sci. Rep.* **7**, 46215; doi: 10.1038/srep46215 (2017).

**Publisher's note:** Springer Nature remains neutral with regard to jurisdictional claims in published maps and institutional affiliations.

## Supplementary Material

Supplementary Information
