# Supplementary material for: Purely one-dimensional bands with a giant spin-orbit splitting: Pb nanoribbons on Si(553) surface
Source: Sci Rep. 2017 Apr 6;7:46215. doi: 10.1038/srep46215 (PMC5382770; doi:10.1038/srep46215)
Supplement: Supplementary Information [file srep46215-s1.pdf]

Supplementary Information: Purely one-dimensional bands with a giant spin-orbit splitting: Pb nanoribbons on Si(553) surface

Marek Kopciuszyński, Mariusz Krawiec, Ryszard Zdyb,* and Mieczysław Jałochowski

Institute of Physics, Maria Curie-Sklodowska University,

Pl. M. Curie-Sklodowskiej 1, 20-031 Lublin, Poland

Additional Figures

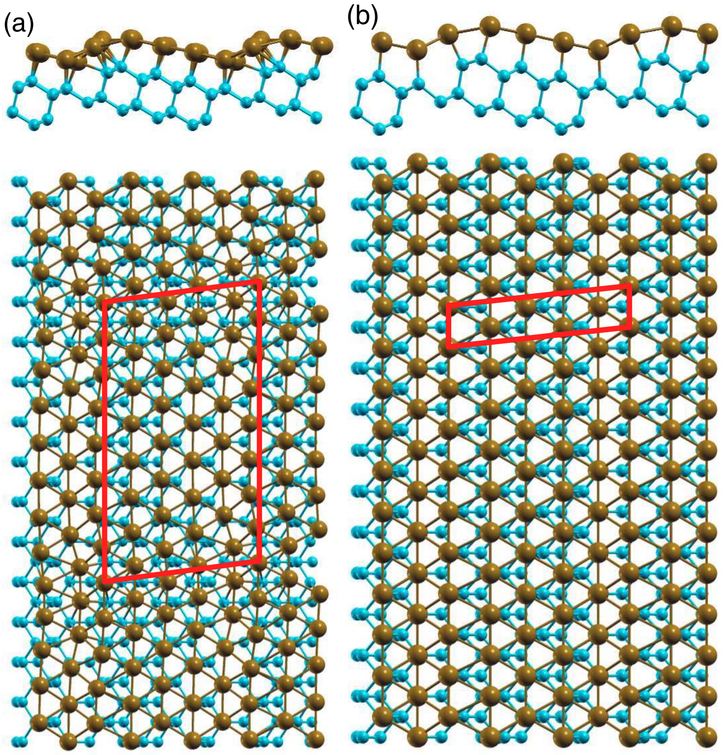

FIG. S1: Structural models of Si(553)-Pb with unit cells outlined in red. (a) The full $7a_{\mathrm{Si}[1\bar{1}0]} \times 1a_{\mathrm{Si}[33\overline{10}]}$ and (b) the reduced $1a_{\mathrm{Si}[1\bar{1}0]} \times 1a_{\mathrm{Si}[33\overline{10}]}$ unit cell geometries. Pb atoms are shown in brown while Si atoms in blue.

*Electronic address: ryszard.zdyb@umcs.pl

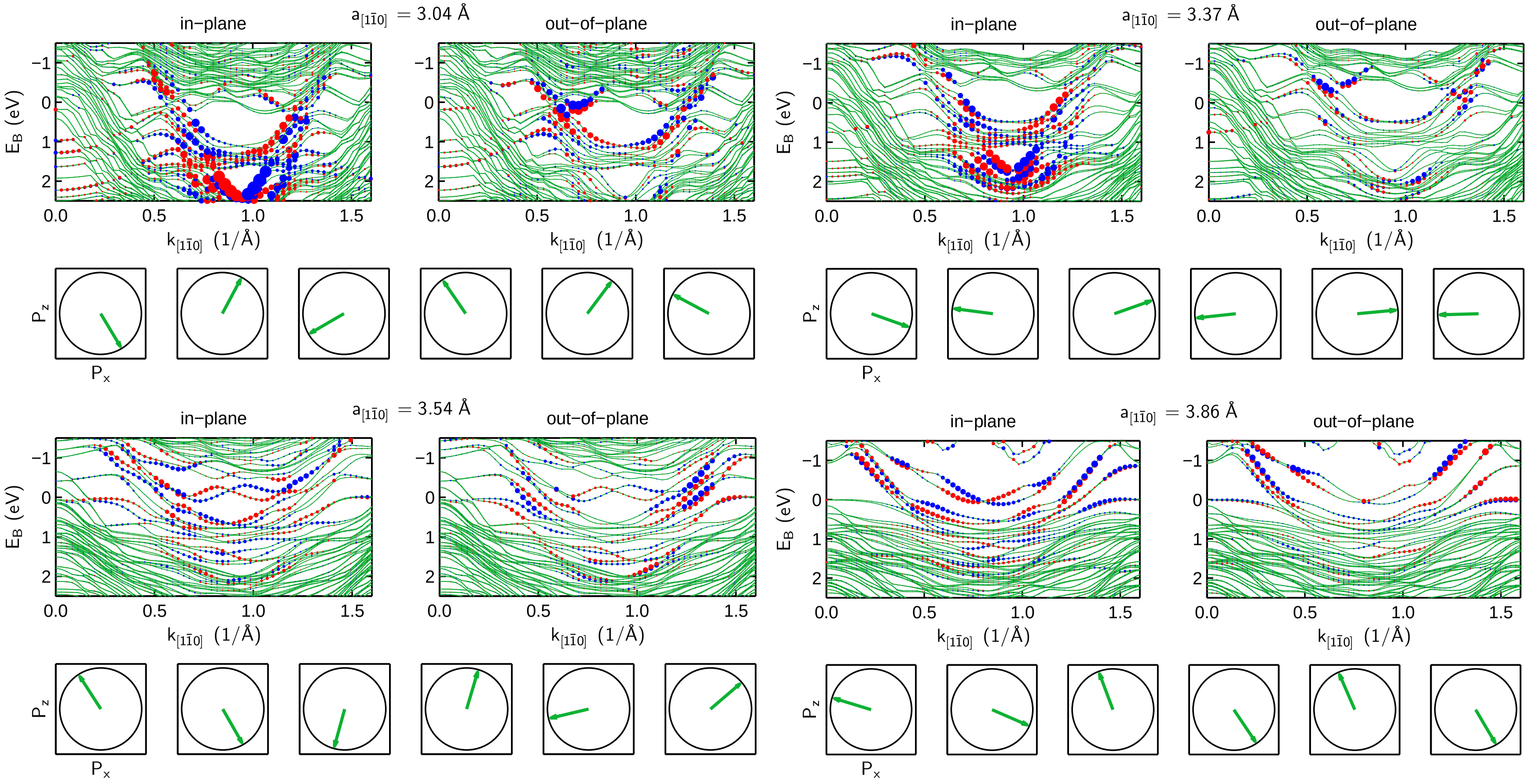

FIG. S2: Band structure of Si(553)-Pb calculated with $a_{[1\bar{1}0]}$ of 3.04, 3.20, 3.37, 3.54 and 3.86 Å. The oppositely polarized bands are indicated by the red and blue circles. A diameter of the circles determines a value of the corresponding component of the polarization vector. Direction of the polarization vector of three pairs of bands determined at $E_B = 0.09$ eV are shown below band structure plots for each lattice constant. The $a_{[1\bar{1}0]} = 3.37$ Å corresponds to the average Pb-Pb distance in Si(553)-Pb system as determined in the STM and RHEED experiments (Kopciuszyński, M. et al. Phys. Rev. B 2013, 88, 155431) and is close to the Pb lattice constant of a freestanding Pb layer. The $a_{[1\bar{1}0]} = 3.04$ Å corresponds to the smallest distance between Pb atoms according to the full unit cell geometry model while $a_{[1\bar{1}0]} = 3.54$ Å and 3.86 Å are inter-atomic distances in the bulk Pb and Si crystals, respectively.

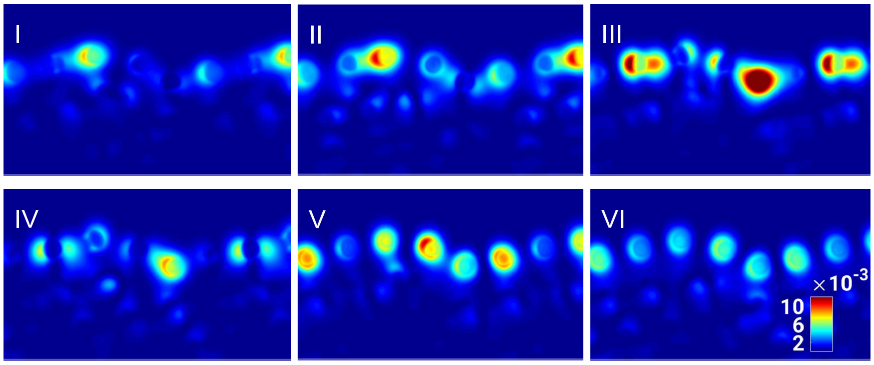

FIG. S3: Partial charge distribution associated with particular bands, indicated by numbers, at $E_B = 0.09$ eV. At that energy the polarization of the spin-split bands has been determined. The charge is calculated for the reduced cell and averaged in the $[1\bar{1}0]$ direction. Positions of Pb atoms are very well visible for band VI as high intensity circles. The color code denotes charge density in electrons/Å^3 units.

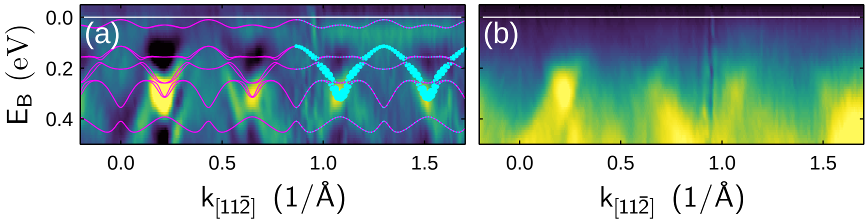

FIG. S4: Photoemission (second derivative (a) and intensity (b)) maps of the binding energy vs wave vector perpendicular to the step edges. Thin solid lines represent results of the DFT calculations. Zero of the binding energy denotes the Fermi level (white line). It is worth to note that intensity of the bands decreases when approaching the Fermi level and the bands become indistinguishable in a high background of the secondary electrons (b). The DFT calculations give similar results - decreasing contribution of the Pb orbitals to the bands which is indicated in (a) by a diameter of the blue circles.

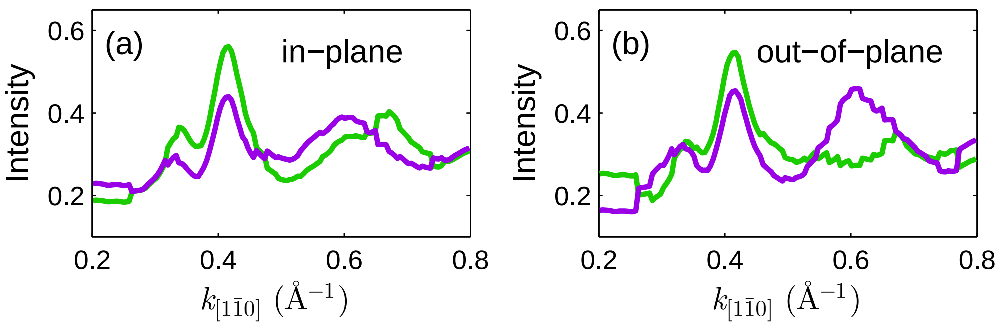

FIG. S5: Spin-resolved photoemission intensity of in-plane (a) and out-of-plane (b) components of the polarization vector.

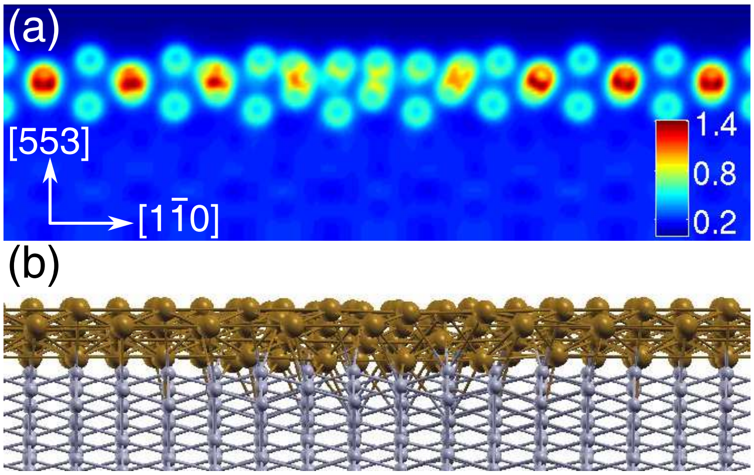

FIG. S6: (a) Calculated charge distribution around Pb atoms of the Si(553)-Pb surface in the full unit cell geometry model. A color code denotes charge density in electrons/bohr^3 units. The charge density is averaged over the whole unit cell along [33$\bar{1}$0]. (b) A side view of the unit cell in the full unit cell geometry model along the [33$\bar{1}$0] direction. The brown (blue) circles denote Pb (Si) atoms.

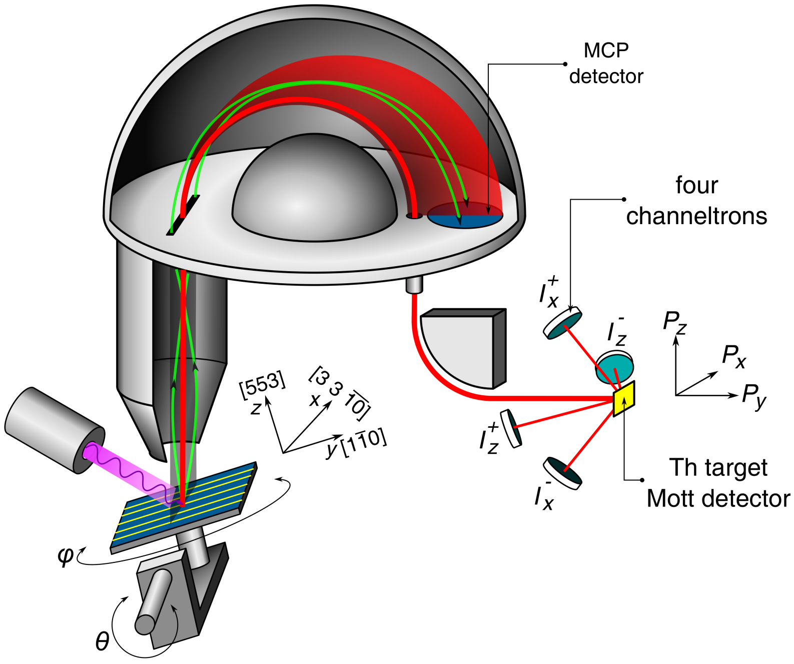

FIG. S7: Geometry of the (S)ARPES experiment.
